# Accurate module induced brain network construction for mild cognitive impairment identification with functional MRI

**DOI:** 10.3389/fnagi.2023.1101879

**Published:** 2023-02-16

**Authors:** Yue Du, Guangyu Wang, Chengcheng Wang, Yangyang Zhang, Xiaoming Xi, Limei Zhang, Mingxia Liu

**Affiliations:** ^1^School of Computer Science and Technology, Shandong Jianzhu University, Jinan, Shandong, China; ^2^School of Mathematics Science, Liaocheng University, Liaocheng, Shandong, China; ^3^School of Computer Science and Cyberspace Security, Hainan University, Haikou, Hainan, China; ^4^Department of Radiology and BRIC, University of North Carolina at Chapel Hill, Chapel Hill, NC, United States

**Keywords:** functional brain network, modularity, Laplacian matrix, mild cognitive impairment, disease identification

## Abstract

**Introduction:**

Functional brain networks (FBNs) estimated from functional magnetic resonance imaging (fMRI) data has become a potentially useful way for computer-aided diagnosis of neurological disorders, such as mild cognitive impairment (MCI), a prodromal stage of Alzheimer's Disease (AD). Currently, Pearson's correlation (PC) is the most widely-used method for constructing FBNs. Despite its popularity and simplicity, the conventional PC-based method usually results in dense networks where regions-of-interest (ROIs) are densely connected. This is not accordance with the biological prior that ROIs may be sparsely connected in the brain. To address this issue, previous studies proposed to employ a threshold or l_1-regularizer to construct sparse FBNs. However, these methods usually ignore rich topology structures, such as modularity that has been proven to be an important property for improving the information processing ability of the brain.

**Methods:**

To this end, in this paper, we propose an accurate module induced PC (AM-PC) model to estimate FBNs with a clear modular structure, by including sparse and low-rank constraints on the Laplacian matrix of the network. Based on the property that zero eigenvalues of graph Laplacian matrix indicate the connected components, the proposed method can reduce the rank of the Laplacian matrix to a pre-defined number and obtain FBNs with an accurate number of modules.

**Results:**

To validate the effectiveness of the proposed method, we use the estimated FBNs to classify subjects with MCI from healthy controls. Experimental results on 143 subjects from Alzheimer's Disease Neuroimaging Initiative (ADNI) with resting-state functional MRIs show that the proposed method achieves better classification performance than previous methods.

## 1. Introduction

Alzheimer's disease (AD), characterized by intellectual disability and abnormal behavior, is the most common form of dementia (Goedert and Spillantini, 2006). With the progress of AD, it will gradually destroy the memory of patients and even affect their ability of daily living. Even though there is no effective treatment for curing AD, the progress of AD disease be delayed by early intervention (Hampel et al., 2008). Thus, classifying the prodromal stage of AD, namely mild cognitive impairment (MCI), has received considerable attention in the past decades (Rombouts et al., 2005; Desikan et al., 2009; Zhu et al., 2014; Jie et al., 2018; Liu et al., 2018; Vogt et al., 2020).

To classify subjects with MCI, researchers have developed different ways for quantitatively measuring the brain activity and organization. Especially, functional brain networks (FBNs) estimated from resting-state functional MRI (rs-fMRI) data have been increasingly employed to study the transition from MCI to AD (Chen et al., 2021). In mathematics, the FBN can be simulated by a graph *G*(*V, E*), where *V* is the node set containing multiple regions-of-interest (ROIs) in the brain, and *E* is the edge set containing the “connections” between pairs of ROIs. In practice, with a given order of the nodes, the FBN can be equivalently described by an edge weighting matrix (i.e., adjacency matrix *W*) (Bullmore and Sporns, 2009; Qiao et al., 2016).

Recent studies have shown that a well-estimated FBN (with *W*) tends to benefit the MCI identification, which makes FBN estimation become a hot research topic in the field (Zhou et al., 2018a; Jiang et al., 2019; Xue et al., 2020). Among various FBN estimation methods developed in the past decades, Pearson's correlation (PC) is the most popular one due to its simplicity and empirical effectiveness. But PC-based methods always result in dense FBNs, which is not accordance with the biological prior of brains (i.e., ROIs may be sparsely connected in the brain) (Bechtel, 2003; Wen et al., 2019). To address this limitation, a threshold is usually utilized to sparsify the estimated FBN by removing weak connections (with edge weights smaller than the pre-defined threshold). Alternatively, Li et al. (2017) introduced an *l*_1_-norm regularizer to the PC model for achieving a sparse FBN.

**Figure 1:**
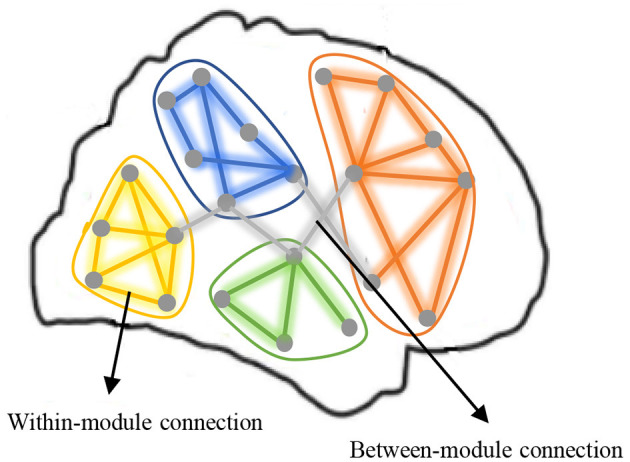
A functional brain network with multiple modules that have dense within-module connection and sparse between-module connection.

It has been reported that FBN generally has more topological structures than just sparsity (Meunier et al., 2009; Zhao et al., 2012; Sporns, 2016; Wang et al., 2019a; Wen et al., 2019). For example, one of the most representative structure is *modularity* that is believed to be extremely important for promoting stability, conserving wiring cost, and enabling complex neuronal dynamics of our brain. As shown in Figure 1, a module in network is a group of nodes with relatively dense interconnections, often corresponding to specialized functional components (Sporns and Betzel, 2016). For FBN, the modular structure can divide the labor of each brain region more clearly, and make our brain work efficiently. To obtain a sparse FBN, some researchers introduced the *l*_1_-norm regularizer to the construction of FBN. The *l*_1_-norm regularization model may automatically find significant network connections and provide sparse solutions since the weights of insignificant connections are automatically driven to zero (Ryali et al., 2012; Jie et al., 2016; Zheng et al., 2018). Specially, Ryali et al. (2012) combined *l*_1_- and *l*_2_-norm regularization for estimating sparse partial correlations between brain regions in fMRI data. Jie et al. (2016) first constructed connectivity hyper-networks from rs-fMRI time series by using *l*_1_-norm to characterize the interactions among different brain regions and then used the hyper-networks for brain disease diagnosis. Zheng et al. (2018) constructed a multi-feature-based network by employing a linear regression model with a *l*_1_-norm penalty to enhance the diagnostic accuracy of AD and MCI and also help discover the underlying neural mechanisms. However, these studies usually ignore modular brain structure, which is an important prior knowledge of the human brain. For instance, the central executive network (CEN) is responsible for high-level cognitive functions such as planning, decision making, and the control of attention and working memory, while the default mode network (DMN) include many brain areas that form an integrated system for self-related mental activity, including autobiographical, self-monitoring, and social functions (Liang et al., 2016).

To address this issue, in this paper, we propose a new FBN estimation method based on graph Laplacian matrix decomposition. Due to the property that the zero eigenvalues of graph Laplacian matrix indicate the connected components (Oellermann and Schwenk, 1991), the proposed method can reduce the rank of the Laplacian matrix to a pre-defined number and obtain FBN with an accurate number of modules. To verify the effectiveness of our method, we use the public Alzheimer's Disease Neuroimaging Initiative (ADNI) dataset to classify subjects with MCI from normal controls (NCs) based on the estimated FBNs. The experimental results show that our method can effectively improve the identification accuracy compared with conventional methods on functional brain network estimation.

The rest of this paper is organized as follows. In Section 2, we introduce the preprocessed data, review three related conventional FBN estimation method, present our proposed method to estimate FBNs and FBN-based brain disease classification. Then, we describe the experimental setting. In Section 3, we report the experimental results on classification tasks and visually compare the FBNs estimated by our AM-PC and six competing methods, and also analyse the influence of module number. In Section 4, we discuss the discmininative features identified by our method and compare our method with several state-of-the-art methods on FBN-based MCI identification with rs-fMRI data from ADNI. Then, we present several limitations of this work and possible future research directions. In Section 5, we summarize the paper.

## 2. Materials and methods

**Figure 2:**
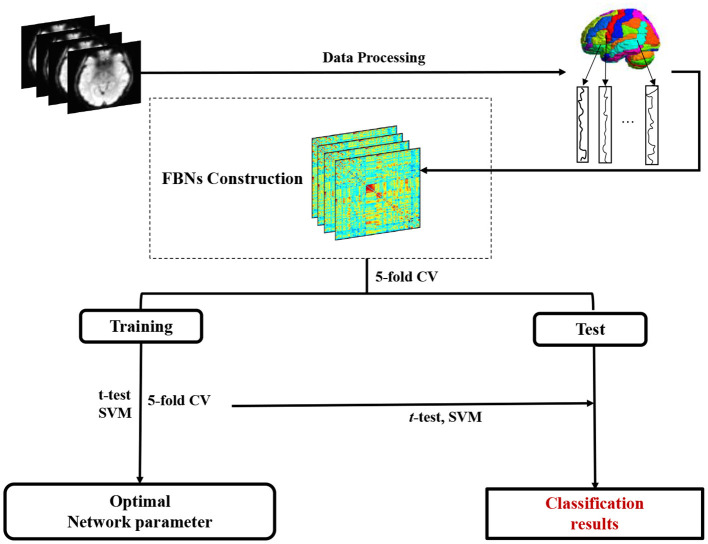
Pipeline of the proposed accurate module induced PC (AM-PC) method for functional brain network (FBN) construction and FBN-based MCI identification with resting-state fMRI data.

As shown in Figure 2, we develop a FBN-based brain disease classification framework, including three major components: fMRI preprocessing, our proposed accurate module induced PC (AM-PC) method for FBN construction, and FBN-based disease classification. In the following, we first introduce the materials and image preprocessing used in this work, and review several PC-based methods. Then, we present the proposed method for FBN estimation and experimental setting.

### 2.1. Materials and image preprocessing

A total of 143 participants from the ADNI dataset (Wang et al., 2019b) were used in this work, including 95 MCIs and 48 NCs. Each participant was scanned at one or more visits in this study, and the interval between two visits is at least 6 months, resulting in a total of 299 resting-state fMRI scans. These 299 scans include 154 NC cases and 145 MCI cases. The scanning parameters were listed as follows. The slice thickness is 3.31 *mm*, TE (echo time) is 30 *ms*, TR (repetition time) is 2.2 − 3.1 *s*, the in-plane image resolution is 2.29 − 3.31 *mm*, and the scanning time for each subject is 7 *min* (resulting in 140 volumes). Table 1 shows the demographic information of these 299 scans.

**Table 1:** Demographic information of the studied 299 *rs*-fMRI scans from the ADNI database.

**Category**	**Scan #**	**Age (Years)**	**Gender (M/F)**
NC	154	75.36 ± 6.16	67/87
MCI	145	71.99 ± 7.67	95/50

The values are denoted as mean±standard deviation. M/F, male/female.

To reduce the influence of nuisance signals, the preprocessing pipeline in FSL FEAT is used in this work. Specifically, for each subject, we discard the first 3 volumes in the fMRI time course for signal stabilization. And then the remaining 137 volumes were processed *via* the standard pipeline. The main steps in preprocessing include slice timing correction, head motion estimation, bandpass filtering, and regression of nuisance covariates (i.e., white matter, cerebrospinal fluid, and motion parameters) by mean regression. Note that subjects were removed if their head motion > 2.0 *mm* of maximal translation or 2.0^*o*^ of maximal rotation. After that, we performed skull stripping based on *T*_1_-weighted MRI and aligned the skull-stripped fMRIs onto the Montreal Neurological Institute space. Then, we used a Gaussian kernel with full-width-at-half-maximum of 6 *mm* to spatially smooth the volumes. The subjects with more than 2.5 *min* of frame-wise displacement (FD > 0.5) were excluded from further analysis. Finally, every brain was divided into 116 ROIs based on the Automated Anatomical Labeling (*AAL*) template (Tzourio-Mazoyer et al., 2002), and the mean time series (with band-pass filtered 0.015 − 0.15 *Hz*) of each ROIs were extracted as the input data for FBN estimation.

### 2.2. Related work

Due to the crucial role in exploring the neurodegenerative diseases, many FBN estimation methods have been proposed in the past decades. In this section, we briefly review several PC-based methods that are closely related to our study.

As pointed out earlier, PC is the simplest and most popular method for FBN estimation (Smith et al., 2013). The edge weight of PC-based method for FBN estimation is defined as follows:


(1) $$\begin{array}{l} w_{ij}=\frac{{\left(x_{i}-{\bar{x}}_{i}\right)}^{T}\left(x_{j}-{\bar{x}}_{j}\right)}{\sqrt{{\left(x_{i}-{\bar{x}}_{i}\right)}^{T}\left(x_{i}-{\bar{x}}_{i}\right)}\sqrt{{\left(x_{j}-{\bar{x}}_{j}\right)}^{T}\left(x_{j}-{\bar{x}}_{j}\right)}}, \end{array}$$

Where $x_{i}\in R^{m}$ is the blood oxygen level-dependent (BOLD) signals associated with the *i*^*th*^ ROI, and $\bar{x_{i}}\in R^{m}$ is a vector containing the mean of the elements in *x*_*i*_.

Without loss of generality, we redefine $x_{i}=(x_{i}-{\bar{x}}_{i})/\sqrt{{\left(x_{i}-{\bar{x}}_{i}\right)}^{T}(x_{i}-{\bar{x}}_{i})}$. Then, Equation (1) can be simplified as $w_{ij}=x_{i}^{T}x_{j}$, which is exactly the solution of the following optimization problem:


(2) $$\begin{array}{ll} \underset{w_{ij}}{\min}\; & \;\overset{n}{\underset{i,j}{\sum }}∥x_{i}-w_{ij}x_{j}|{|}^{2}, \end{array}$$

or its matrix form shown as follows:


(3) $$\begin{array}{ll} \underset{W}{\min}\; & \;||W-X^{T}X|{|}_{F}^{2}, \end{array}$$

Where *W* is the adjacency matrix to be estimate by PC, $X=\left[x_{1},x_{2},\cdots \;,x_{n}\right]\in R^{m\times n}$ is the data matrix containing the fMRI time courses, and *n* is the number of ROIs. We will note shortly that such an optimization view of PC can help us to develop new and more flexible FBN estimation methods.

Despite its popularity, PC aims to measure the full correlation between signals of all ROIs in the brain, thus generating dense networks where all nodes/ROIs are densely connected. This is not consistent with the empirical finding that sparsity has been proven to be the most popular property of FBN (Sporns, 2016). Therefore, in practice, a threshold is generally used to sparsify the originally estimated FBN by removing the edges with weak weights. An alternative to sparsify FBN is the *l*_1_-regularized PC (PC_*Sparsity*_) (Li et al., 2017) whose model is given as follows:


(4) $$\begin{array}{l} \underset{W}{\min}\;||X-XW|{|}_{F}^{2}+\lambda ||W|{|}_{1}, \end{array}$$

where ||*W*||_1_ is the *l*_1_-norm of a matrix for encoding the sparsity prior of FBN, and λ is a regularized parameter for controlling the sparsity of *W*.

Besides sparsity, FBNs usually have more richer structures (Sporns, 2016) such as modularity, and some of these structures may guide us to estimate more reasonable FBNs. Recently, Zhou et al. (2018b) developed an M-FBN method to estimate FBN by further introducing a trace norm regularizer into Equation (4), resulting in the following model:


(5) $$\begin{array}{l} \underset{W}{\min}\;||X-XW|{|}_{F}^{2}+\lambda_{1}||W|{|}_{1}+\lambda_{2}||W|{|}_{*}, \end{array}$$

Where ||*W*||_*_ is the trace norm of the matrix *W*, and λ_1_ and λ_2_ are regularized parameters for controlling the balance between the three terms in the objective function. With the combination of *l*_1_ and trace norms, Equation (5) has been verified to be able to discover modular structures of the estimated FBN. However, since this model is an approximation of the sparse and low-rank matrix, the network estimated by M-FBN could not include a notable modular structure.

### 2.3. Proposed method for FBN estimation

Several previous Studies (Grone et al., 1990; Zhou et al., 2018b) proposed to jointly minimize the *l*_0_-“norm” and the rank of the edge weighting matrix *W*, since a sparse (*via* minimizing *l*_0_-“norm”) and low-rank (*via* minimizing the rank) matrix tends to result in modular structures. However, these two regularizers are both non-convex with respect to *W*, making the optimization problem intractable. In practice, they are generally relaxed to *l*_1_-norm ||*W*||_1_and trace norm ||*W*||_*_ respectively. Such relaxation only achieves an approximation of a sparse low-rank matrix, and thus fails to guarantee the modular structure of FBN.

Motivated by the theorem (Heider, 1946) that the multiplicity *k* of the eigenvalues 0 of a Laplacian matrix is equal to the number of connected components in a graph, we propose a new method to estimate FBNs with an accurate number of modularity. Denote *n* (*n* = 116 in this work) as the number of ROIs and *k* (*k* = 8 in this work) as the number of modules. Specifically, based on the *l*_1_-regularized PC method defined in Equation (4), we propose to constrain the rank of *L*_*W*_ as *n*−*k* to generate a FBN with an accurate number of modules. Mathematically, the proposed **AM-PC** model is given as follows:


(6) $$\begin{array}{l} \begin{array}{cc} \underset{W}{\min}\; & \;\|W-X^{T}X{\|}_{F}^{2}+\lambda \Sigma_{i,j}^{n}\|W{\|}_{1}\\ s.t.\; & \;W\geq 0,\;rank\left(L_{W}\right)=n-k, \end{array} \end{array}$$

Due to the nonnegative constraint in Equation (6), the *l*_1_-norm can be replaced by the sum of the elements in the matrix. In fact, the nonnegative assumption for edge weights is supported by the structural equilibrium theory (Fan, 1949; Cartwright and Harary, 1956), and can simplify the subsequent analysis for FBNs. With Equation (6), we can explicitly construct an FBN with a total of *k* modules for each subject.

Given the data matrix *X* and a matrix *A* = *X*^*T*^*X*, we can rewritten Equation (6) as follows:


(7) $$\begin{array}{l} \begin{array}{cc} \underset{W}{\min}\; & \;\|W-A{\|}_{F}^{2}+\lambda \Sigma_{i,j}^{n}W_{ij}\\ s.t.\; & \;W\geq 0,\;rank\left(L_{W}\right)=n-k. \end{array} \end{array}$$

In general, it is not straightforward to solve (Equation 7) since *rank*(*L*_*W*_) = *n*−*k* is a strict constraint. In what follows, we derive an efficient optimization algorithm to solve this challenging problem.

Denote σ_*i*_(*L*_*W*_) as the *i*-th smallest eigenvalue of *L*_*W*_. Accordingly, Equation (7) can be equivalently converted to the following problem:


(8) $$\begin{array}{l} \underset{W\geq 0}{\min}\;||W-A|{|}_{F}^{2}+\alpha \Sigma_{i=1}^{k}\sigma_{i}\left(L_{W}\right)+\lambda \Sigma_{i,j}^{n}W_{ij}, \end{array}$$

Where *L*_*W*_ is positive semi-definite to guarantee σ_*i*_(*L*_*W*_)≥0, and a large α enables $\Sigma_{i=1}^{k}\sigma_{i}\left(L_{W}\right)=0$ to meet the constraint of *rank*(*L*_*W*_) = *n*−*k*.

Furthermore, our proposed optimization method is based on the Ky Fan's theorem (Grant and Boyd, 2014) as follows:


(9) $$\begin{array}{l} \Sigma_{i=1}^{k}\sigma_{i}\left(L_{W}\right)=\underset{F\in R^{n\times k},F^{T}F=I}{\min}Tr\left(F^{T}L_{W}F\right). \end{array}$$

By combining Equations (8), (9), we have the problem:


(10) $$\begin{array}{l} \begin{array}{cc} \underset{W\geq 0}{\min}\; & \;||W-A|{|}_{F}^{2}+\alpha Tr\left(F^{T}L_{W}F\right)+\lambda \Sigma_{i,j}^{n}W_{ij}\\ s.t.\; & W\geq 0,\;F\in R^{n\times k},\;F^{T}F=I. \end{array} \end{array}$$

Which can be efficiently solved by the following alternating optimization algorithm.

**Step 1**: When *W* is fixed, Equation (10) becomes


(11) $$\begin{array}{l} \underset{F\in R^{n\times k},F^{T}F=I}{\min}Tr\left(F^{T}L_{W}F\right), \end{array}$$

Whose the optimal solution is formed by the *k* eigenvectors corresponding to the *k* smallest eigenvalues of *L*_*W*_.

**Step 2**: When *F* is fixed, Equation (10) becomes


(12) $$\begin{array}{l} \underset{W\geq \;0}{\min}\;||W-A|{|}_{F}^{2}+\alpha Tr\left(F^{T}L_{W}F\right)+\lambda \overset{n}{\underset{i,j}{\sum }}W_{ij}, \end{array}$$

which is equivalent to the following problem:


(13) $$\begin{array}{l} \underset{W\geq \;0}{\min}\;\underset{i,j}{\sum }{\left(W_{ij}-a_{ij}\right)}^{2}+\frac{\alpha }{2}\underset{i,j}{\sum }||f_{i}-f_{j}|{|}_{2}^{2}W_{ij}+\lambda \overset{n}{\underset{i,j}{\sum }}W_{ij}. \end{array}$$

For simplicity, we denote $||f_{i}-f_{j}|{|}_{2}^{2}$ as *f*_*ij*_. Then, by expanding and combining the like terms, Equation (13) can be rewritten as follows:


(14) $$\begin{array}{l} \underset{W\geq 0}{\min}\;\underset{i,j}{\sum }\left(W_{ij}^{2}+\left(\frac{\alpha }{2}f_{ij}-2a_{ij}+1\right)W_{ij}+a_{ij}^{2}\right), \end{array}$$

Which is a quadratic programming problem and can be easily solved by, for example, the CVX toolbox (Grant and Boyd, 2014). We summarize the optimization algorithm to solve Equation (10) in Algorithm 1.

**Algorithm 1:** Algorithm for solving the proposed model in Equation (10).

**Require:** Data Matrix *X*, the number of modules *k*, the parameters λ and α.
**Ensure:** Edge weight matrix *W*.
1: Update *F*, the optimal solution of *F* is composed of *k* eigenvectors corresponding to the *k* smallest eigenvalues of *L*_*W*_.
2: Update *W*, the optimal solution of *W* is obtained by solving problem (15).

### 2.4. Experimental setting

Based on the learned edge weighting matrix *W*, we can construct a specific FBN with *k* modules for each subject. Given FBNs for all subjects, the subsequent task is to classify MCI and NC based on the estimated FBNs. Since the given FBNs are symmetry matrices, the upper and lower triangle matrix features are the same, and for ease of calculation, we take the upper triangle features including (116 × (116 − 1))÷2 = 6, 670 elements. Although the edge weight matrix contains the full information of the network, it typically causes the curse of dimensionality, since the number of feature dimension (Kuo and Sloan, 2005), *i.e*. 6670, is far greater than the number of subjects.

To address this issue, we propose to employ the *t*-test algorithm to select the most informative features by a fixed *p* values (*p* = 0.05 in this work). As pointed out in Wee et al. (2014), the classifier design has a big influence on the ultimate accuracy. For this reason, we employ a linear support vector (SVM) with the default parameter (i.e., *C* = 1) as classifier (Chang and Lin, 2011) in this work, considering that it is simple and widely used in neuroimaging-based brain disorder classification.

In the experiments, we use a 5-fold *subject-level* cross-validation (CV) strategy (Li et al., 2017) to evaluate different methods, to ensure that fMRI scans of the same subject will not appear in both training and testing sets. To reduce the biased introduced by random partition cross validation, the 5-fold CV process was repeated 100 times for all methods. Besides, for the fair comparison, all the competing methods use the same linear SVM (with *C* = 1) as the classifier.

Since the parameters may significantly affect the structure of the constructed networks and the classification results, we select optimal parameters through grid search *via* inner 5-fold CV based on only training data. Specifically, we uniformly utilize 11 candidate values [2^−5^, 2^−4^, ⋯ , 2^5^] for the regularization parameters (λ, λ_1_ and λ_2_) in the four competing methods (i.e., PC_*Sparsity*_, PC_*Sparsity*_+, M-PC, and M-PC+). The proposed AM-PC has three parameters, i.e., *k*, λ and α. The module number *k* is empirically set as 8 based on the prior knowledge (Wong, 2015). The optimal value of α can be determined by a heuristic approach. That is, we first initialize α with a small value. Then, in each iteration, we compute the number of zero eigenvalues in *L*_*W*_, if it is larger than *k*, then divide α by 2; if it is smaller than *k*, then multiply α by 2; otherwise stop the iteration. Thus, in AM-PC, we only need to tue the parameter λ. In our experiment, the elements in the network tend to zero when λ is equal to the number in [2^0^, 2^1^, ⋯ , 2^5^], and the network loses its discriminative capability. Therefore, the optimal value of λ in our AM-PC is selected from [2^−11^, 2^−10^, ⋯ , 2^−1^] *via* inner 5-fold CV.

## 3. Results

With the extracted mean signal of each ROI, we estimate FBNs *via* the proposed **AM-PC** method and three different methods, including (1) **PC** with its model defined in Equation (3), (2) **PC**_***Sparsity***_ (Li et al., 2017) with its model defined in Equation (4), and (3) **M-PC** (Zhou et al., 2018b) with its model defined in Equation (5). The proposed AM-PC usually selects an ROI with small weights and limited degrees as a separate module. To alleviate this problem, we use the normalized Laplacian matrix *L*_*W*_ instead of the original Laplacian matrix. In the proposed AM-PC, the constraint in Equation (6) helps generate a nonnegative FBN for each subject. For a fair comparison, we also compare our AM-PC with three additional methods, including (1) **PC+**, (2) **PC**_***Sparsity***_**+**, and (3) **M-PC+**, and these three methods remove the negative edges in networks estimated by PC, PC_*Sparsity*_, and M-PC, respectively.

### 3.1. Classification results

In this section, we perform MCI identification (i.e., MCI vs. NC classification) based on the FBNs estimated by seven different methods (including PC, PC+, PC_*Sparsity*_, PC_*Sparsity*_+, M-PC, M-PC+, AM-PC). For seven methods, we employ the same *t*-test algorithm for feature selection and the linear SVM for classification to ensure the fair comparison. Four metrics are used to evaluate the classification performance, including accuracy (ACC), sensitivity (SEN), specificity (SPE) and AUC. Denote TP, TN, FP and FN as true positive, true negative, false positive and false negative, respectively. These four metrics are defied as follows: ACC=$\frac{TP+TN}{TP+FP+TN+FN}$, SEN=$\frac{TP}{TP+FN}$, SPE=$\frac{TN}{TN+FP}$, and AUC is the area under the ROC (receiver operating characteristic) curve.

In Table 2, we report the classification results of MCI vs. NC classification achieved by seven different ways. The term marked by “*” denotes that the result of proposed AM-PC is significantly better than that of all six competing methods (with *p* < 0.05). It can be seen from this table that our proposed AM-PC method consistently outperforms the six competing methods in terms of four evaluation metrics. And the AM-PC is significantly better (with *p* < 0.05) than the six methods in terms of ACC, SPE and AUC values. These results imply that the modularity prior introduce by our method plays an important role in modeling the rich topological structure of functional brain networks, thus helping boost the identification performance of subjects with MCI.

**Table 2:** Classification performance (mean ± standard deviation) of 7 different methods in MCI vs. NC classification.

**Method**	**ACC**	**SEN**	**SPE**	**AUC**
PC	0.7454 ± 0.0028	0.7198 ± 0.0128	0.7730 ± 0.0081	0.8246 ± 0.0083
PC+	0.7692 ± 0.0115	0.7847 ± 0.0151	0.7551 ± 0.0167	0.8563 ± 0.0662
PC_*Sparsity*_	0.7705 ± 0.0100	0.7386 ± 0.0113	0.7995 ± 0.0120	0.8475 ± 0.0061
PC_*Sparsity*_+	0.7899 ± 0.0099	0.7820 ± 0.0114	0.8035 ± 0.0303	0.8728 ± 0.0036
M-PC	0.7801 ± 0.0111	0.7727 ± 0.0228	0.8046 ± 0.0180	0.8352 ± 0.0056
M-PC+	0.7837 ± 0.0102	0.7805 ± 0.0206	0.7861 ± 0.0045	0.8616 ± 0.0024
AM-PC (Ours)	**0.8013 ± 0.0106** ^*^	**0.7836 ± 0.0130**	**0.8182 ± 0.0189** ^*^	**0.8773 ± 0.0075** ^*^

The term marked by “*” denotes that the result of proposed AM-PC is significantly better than that of all six competing methods (with *p* < 0.05).

The bold values indicate the best results.

### 3.2. Estimated functional brain networks

**Figure 3:**
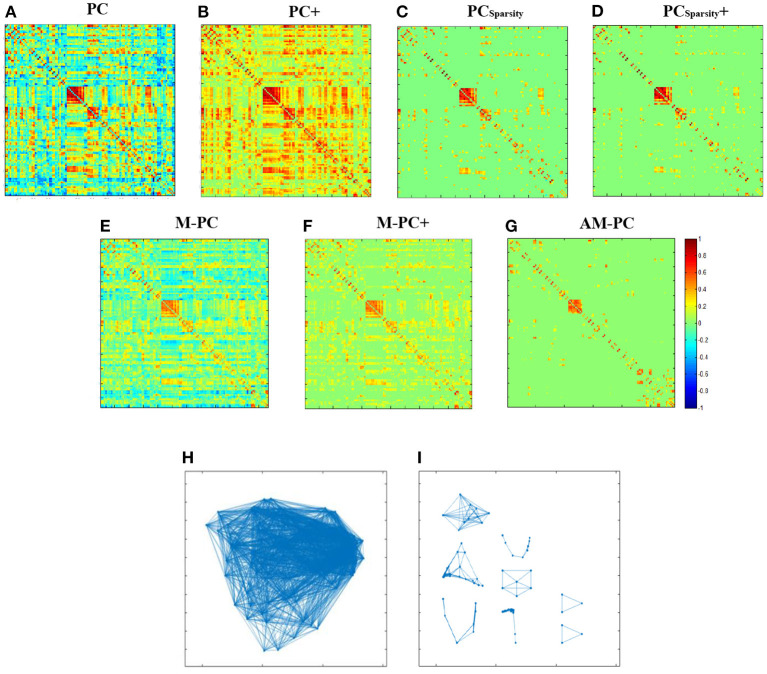
Seven edge weight matrices of the same subject estimated by seven different methods, i.e., **(A)** PC, **(B)** PC+, **(C)** PC_*Sparsity*_, **(D)** PC_*Sparsity*_+, **(E)** M-PC, **(F)** M-PC+, and **(G)** AM-PC. **(H, I)** show the modules in the networks estimated by M-PC and the proposed AM-PC methods.

We visually compare the FBNs estimated by our AM-PC and six competing methods. In Figures 3A–G, we take a randomly selected subject from ADNI as an example to visualize the FBNs estimated by these seven methods. Here, the corresponding parametric values are λ = 2^−2^ for PC_*Sparsity*_ and PC_*Sparsity*_+, $\lambda_{1}=2^{3}$, $\lambda_{2}=2^{2}$ for M-PC and M-PC+, and λ = 2^−1^ for our AM-PC.

It can be seen from Figures 3A–G that the FBNs estimated by PC_*Sparsity*_, PC_*Sparsity*_+, M-PC, M-PC+ and AM-PC are sparse. This is due to the introduction of the *l*_1_-norm regularizer in these three methods. Besides, the FBN estimated by our proposed AM-PC method shows clearer modular structure than others. The underlying reason could be that AM-PC apply a low-rank constraint to *L*_*W*_, thus yielding more clear modules in the estimated functional brain network.

To show the modular structure of the network constructed by AM-PC more clearly, we use scatter plots to illustrate the networks estimated by M-PC and our AM-PC in Figures 3H, I, respectively. From Figures 3H, I, we can observe that our AM-PC can generate an accurate number (i.e., *k* = 8) modules in the estimated FBN, compared with the M-PC method that aims to generate FBNs with approximate modules. These results further validate the effectiveness of the proposed method in generating FBNs with clear modular structures.

### 3.3. Influence of number of modules

**Figure 4:**
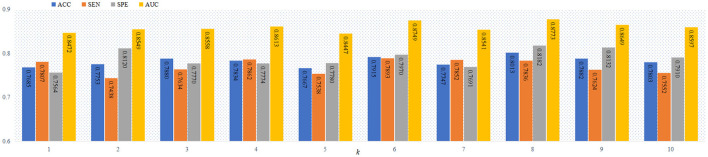
The ACC, SEN, SPE and AUC values of our AM-PC method with different numbers of modules (i.e., *k*) in the task of MCI vs. NC classification.

As a complex network, FBN include the hierarchical structure (Meunier et al., 2009, 2010), and the number of modules increase with the deeper of the FBN level. Thus, it is a practical problem to select the optimal values of the parameter *k* in the proposed objective function in Equation (6). Previous studies works (Bertolero et al., 2015; Geerligs et al., 2015; Gallen et al., 2016; Murakami et al., 2018) have shown that the number of modules in the FBN is usually less than 10. To study the influence of the number of modules on the classification performance, we report the four performance metrics (i.e., ACC, SEN, SPE and AUC) achieved by the proposed AM-PC method using different values of *k* in Figure 4. From Figure 4, we can see that the AM-PC method can achieve overall stable results when 6 ≤ *k* ≤ 9, and the best AUC value is achieved with *k* = 8.

## 4. Discussion

In this section, we visually show the most discriminative features and modular structures identified by our method. We also compare our method with several state-of-the-art methods and list several limitations of the current work as well as possible future research directions.

### 4.1. Discriminative functional connections

**Figure 5:**
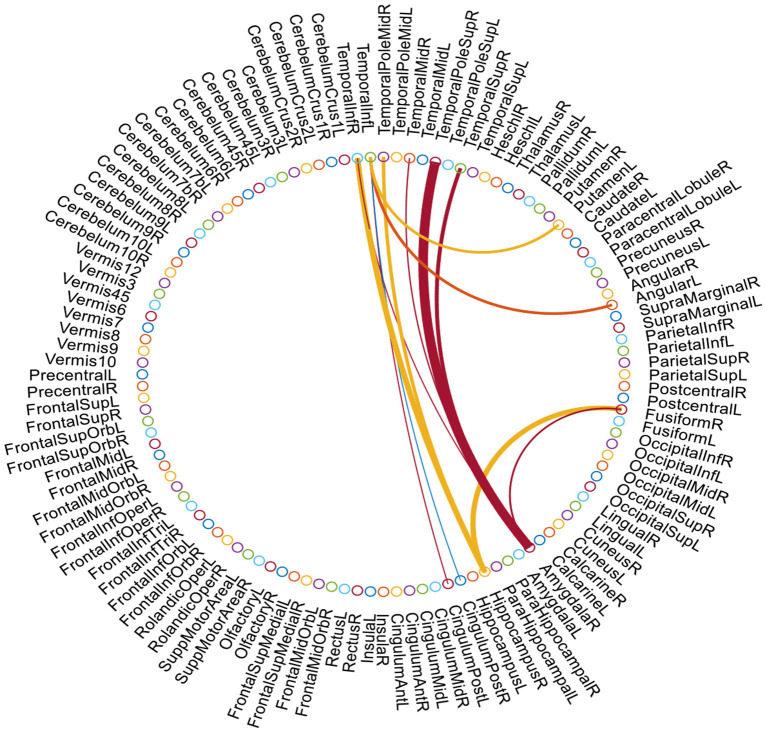
Top 12 discriminative connections and ROIs in our estimated FBNs selected by *t*-test. The thickness of each arc denotes the discriminative power of the corresponding connection in MCI vs. NC classification.

We further show the most discriminative functional connections in FBNs estimated by the proposed AM-PC method. We empirically set *k* = 8 and use the *t*-test (with *p* = 10^−6^) to select the top 12 most informative connections. The identified discriminative connections are shown in Figure 5, where the discriminating power of a connection between two ROIs is represented by the thickness of an arc. As can be seen from Figure 5, the discriminative brain regions corresponding to these selected connections include several important ROIs, such as *right hippocampus, right amygdala*, and *middle temporal gyrus*. Especially, a clear discriminative functional connectivity exists between right temporal pole sup and right amygdala, and such connectivity plays an important role in cognition and emotion (Menon, 2018). The discriminative ROIs identified by our method also include right hippocampus, a brain region that is primarily associated with memory (Disouky et al., 2022). This finding is consistent with previous research (Albert et al., 2011; Kesler, 2014; Zhu et al., 2015), which further validates the effectiveness of our AM-PC method in constructing reliable functional brain networks for MCI identification.

### 4.2. Identified modular structure

**Figure 6:**
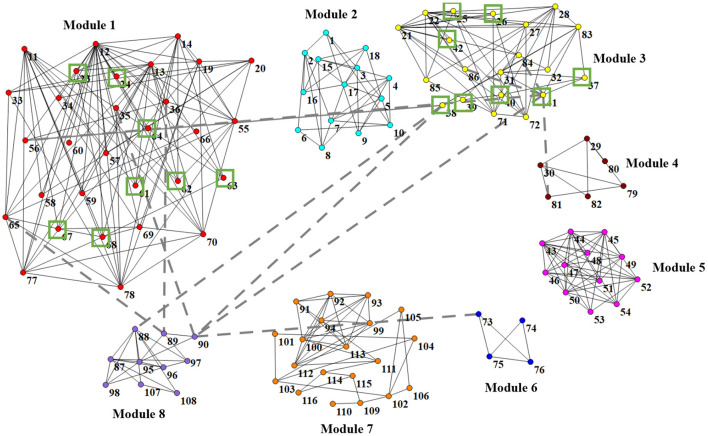
Identified 8 modules of the average network (among training subjects), where nodes with the same color belong to the same module. Gray dotted lines represent the 12 most discriminative connections. Green boxes denote 16 brain regions that are related to MCI (Suk et al., 2015; Yu et al., 2017).

**Figure 7:**
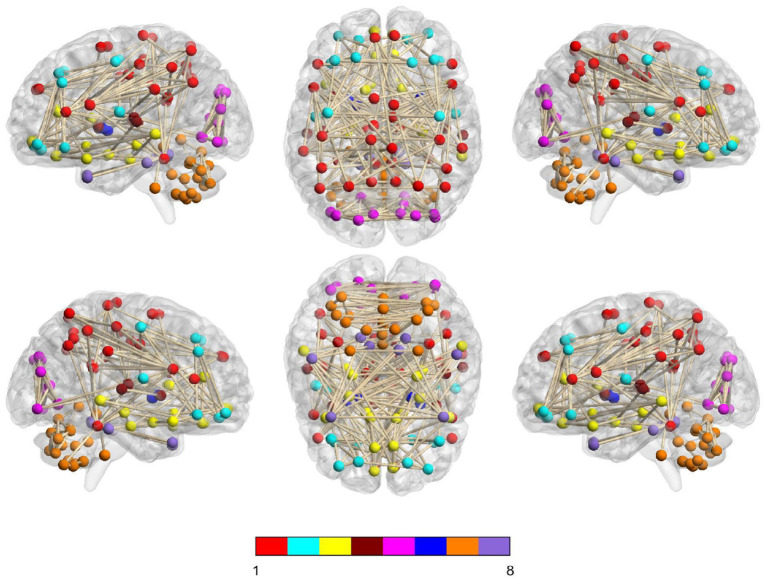
Identified 8 modules of the average network (among training subjects) on the brain template, where nodes with the same color belong to the same module.

In Figure 6, we visually show the modular structure of the average functional brain network (i.e., the average FBN of all training subjects), including 8 modules (with α = 2^−3^). The gray dotted lines represent the 12 most discriminative connections identified by our AM-PC method (see Figure 6). In the following Figure 7, we map Figure 6 on the brain template through BrainNet Viewer (Xia et al., 2013), where each node is a brain region, and each edge is the link between the brain region.

From Figures 6, 7, we have the following observations. *First*, we can clearly see that these eight modules are sparsely connected with each other, while nodes/ROIs within each module are densely connected, which caters to the results in previous papers (Meunier et al., 2010; Bertolero et al., 2018). *Second*, the ROIs contained in the *Module* 1 and *Module* 3 are associated with cognitive functions of the brain, involving the middle temporal gyrus (ROI IDs: 85 and 86), hippocampus (IDs: 37 and 38), parahippocampus (IDs: 39 and 40), precuneus (IDs: 67 and 68), amygdalae (IDs: 41 and 42), supramarginal gyrus (IDs: 63 and 64), inferior parietal lobules (IDs: 61 and 62), superior-medial frontal gyrus (IDs: 23 and 24), and medial orbitofrontal gyrus (IDs: 25 and 26). These regions are believed to be biologically associated with MCI, as reported in previous studies (Yetkin et al., 2006; He et al., 2007; Fair et al., 2008). *Besides*, we found that most of the 12 most discriminative connections are distributed between *Module* 1, *Module* 2 and *Module* 3. These results may imply that the interruption of the connections between the three modules could be used as potential biomarkers for MCI detection.

### 4.3. Comparison with state-of-the-arts

We also compare our AM-PC method with several state-of-the-art methods on FBN-based MCI identification with rs-fMRI data from ADNI. In Table 3, we briefly summarize the classification results of several previous studies as well as our method, where the top 2 best results are shown in bold. As can be seen from Table 3, our AM-PC method can achieve the overall comparable results compared with four SOTA methods. Even though the ACC and AUC results reported in Yang et al. (2019) are better than ours, their evaluation was based on a relatively smaller dataset compared with this work.

**Table 3:** Comparison with state-of-the-art methods for FBN-based MCI vs. NC classification with rs-fMRI data from ADNI.

**Method**	**Subject #**	**ACC**	**SEN**	**SPE**	**AUC**
Chen et al. (2017)	54MCI+54NC	0.7870	0.7778	**0.7963**	0.8449
Kam et al. (2019)	49MCI+48NC	0.7607	0.7627	0.7587	−
Yang et al. (2019)	47MCI+29NC	**0.8298**	0.7662	−	**0.9406**
Xue et al. (2020)	45MCI+46NC	0.7692	**0.8222**	0.7174	−
AM-PC (Ours)	95MCI+48NC	**0.8013**	**0.7836**	**0.8182**	**0.8773**

The top 2 best results are shown in bold.

### 4.4. Limitations and future work

The current work has some limitations. *First*, in this work, we obtained the modularity of FBNs based on the PC-based networks. In fact, our method can also be combined with other methods (such as sparse representation) to estimate FBNs with modularity (Qiao et al., 2018), which will be our future work. *Second*, we focus on the within-module connection in this work by introducing an accurate number of modules in the estimated FBNs, without emphasizing between-module connections. In the future, we plan to incorporate both within-module and between-module connections in to the proposed framework for FBN-based brain disease analysis. *Third*, the small sample size could be an important limitation to the generalizability and replicability of this study. To alleviate this problem, we will utilize transfer learning (Pan and Yang, 2010; Valverde et al., 2021) or meta-learning (Finn et al., 2017; Hospedales et al., 2021) strategy to model the modularity structure of FBN.

## 5. Conclusion

In this paper, we propose AM-PC method to estimate FBNs for MCI identification. Specifically, we explicitly impose constraints on the rank of the Laplacian matrix and the number of modules of the brain network, aiming to construct sparse FBNs with an accurate number of modules. We also develop an effective optimization algorithm to solve the proposed objective function. Experiments were performed on the ADNI dataset, and the AM-PC achieves the accuracy of 80.13%, sensitivity of 78.13%, specificity of 81.82%, and AUC of 87.73% in MCI identification.

## Data availability statement

The original contributions presented in the study are included in the article/supplementary material, further inquiries can be directed to the corresponding author.

## Author contributions

YD: conceptualization, methodology, data curation, writing-original draft, writing-review, and editing. GW: methodology, data curation, investigation, writing-review, and editing. CW and YZ: methodology and data curation. XX: writing-review, editing, and supervision. ML and LZ: conceptualization, resources, and supervision. All the authors read and approved the final manuscript.
